# Intramyometrial injection versus intravenous infusion of oxytocin for maintaining uterine contractility during elective caesarean delivery in a randomised controlled trial

**DOI:** 10.1038/s41598-026-46727-z

**Published:** 2026-03-31

**Authors:** Satoshi Naruse, Chieko Akinaga, Yusuke Mazda, Yoshiki Nakajima

**Affiliations:** 1 Perinatal Center, Hamamatsu University School of Medicine, 1-20-1 Handayama, Chuo-ku, Hamamatsu, Shizuoka 431-3192 Japan; 2 Department of Anesthesiology and Intensive Care, Hamamatsu University School of Medicine, 1-20-1 Handayama, Chuo-ku, Hamamatsu, Shizuoka 431-3192 Japan; 3 Department of Obstetric Anesthesiology, Center for Maternal-Fetal and Neonatal Medicine, Saitama Medical Center, Saitama Medical University, 1981 Kamoda, Kawagoe, Saitama 350-8550 Japan

**Keywords:** Caesarean delivery, Haemodynamic stability, Intramyometrial oxytocin, Intravenous oxytocin, Postpartum haemorrhage, Uterine contractility, Medical research, Signs and symptoms

## Abstract

Oxytocin is commonly administered during caesarean delivery (CD) to prevent postpartum haemorrhage as an initial bolus followed by a maintenance dose. Nearly half of perinatal centres in Japan utilise intramyometrial (IMY) oxytocin. IMY oxytocin is less effective than intravenous (IV) oxytocin as the initial dose for inducing uterine contractions. However, its role as a maintenance dose remains unclear. We evaluated the efficacy of IMY oxytocin versus continuous IV infusion for maintaining uterine contractility during elective CD. Twenty-two women undergoing elective CD were randomised to receive an initial IV bolus of 1 IU of oxytocin, followed by either 5 IU IMY oxytocin or a continuous IV infusion of 5 IU oxytocin. Total blood loss, along with haemodynamic parameters, was assessed. No significant difference in total blood loss was observed between the IMY and IV groups (median 634 mL [540–718] vs. 642 mL [507.5–706.5], P = 0.66). Conversely, IMY oxytocin resulted in smaller reductions in blood pressure and lower phenylephrine requirements after delivery. While IMY oxytocin did not significantly reduce total blood loss compared with continuous IV infusion, it was associated with better haemodynamic stability. Further studies are required to confirm these findings.

*Clinical trial registration*: Japan Registry of Clinical Trials, jRCTs041210153, 25/04/2022.

**Supplementary Information:**

The online version contains supplementary material available at 10.1038/s41598-026-46727-z.

## Introduction

Postpartum haemorrhage (PPH) is a leading cause of maternal mortality and morbidity globally^1,2^, making it crucial to mitigate its incidence and severity. Oxytocin is the first-line medication for the prevention and treatment of PPH, and the World Health Organization recommends its prophylactic use for all deliveries^3^. Despite its widespread use and effectiveness, oxytocin administration can cause adverse events, such as nausea, vomiting, hypotension, tachycardia, ST segment changes^4,5^, and even maternal death^6^. These adverse effects are dose-dependent^7,8^. Consequently, an international consensus statement recommends administration of the minimum effective dose of oxytocin to minimise adverse reactions^9^.

Oxytocin is typically administered in two phases to prevent PPH: an initial dose to induce uterine contractions^10,11^ and a maintenance dose to sustain them^5,12^. Oxytocin has a short half-life of 3–5 minutes^13^. Studies have suggested that adding a maintenance dose after the initial dose can reduce bleeding as well as the need for additional uterotonics^14,15^.

Several routes of oxytocin administration exist, including intravenous (IV), intramuscular, and intramyometrial (IMY). Of these routes, IV administration is preferred in many situations because of its rapid onset and reliable delivery of both the initial and maintenance doses^9^. In contrast, IMY administration is frequently used during caesarean delivery (CD) in some countries, including Japan^16–18^, likely because it is expected to achieve localised effects by injecting oxytocin directly into the uterine muscle and because obstetricians prefer to administer oxytocin directly in the surgical field.

Our previous study showed that IMY administration of oxytocin was less effective than IV administration for achieving rapid uterine contractions^19^. Nevertheless, a recent survey targeting obstetricians revealed that nearly half of the perinatal centres in Japan continue to use IMY oxytocin^18^, suggesting a perceived benefit of its use beyond the initial dose. We speculate that obstetricians may favour IMY oxytocin because of its potential utility in maintaining uterine contractility. However, its role as a maintenance dose has not been fully evaluated. Therefore, we hypothesised that IMY injection would be more effective than continuous IV infusion for maintaining the uterine contractile effects of oxytocin, and conducted this trial to evaluate this hypothesis.

## Methods

### Study design and ethics

This double-blind, single-centre, randomised trial with a 1:1 allocation ratio compared IMY injection with continuous IV infusion of oxytocin as a maintenance dose during elective CD under neuraxial anaesthesia. The study was approved by the Clinical Research Review Board of the Hamamatsu University School of Medicine (Protocol C023-2021), and all participants provided written informed consent. The study was conducted at Hamamatsu University Hospital and registered in the Japan Registry of Clinical Trials (jRCT), jRCTs041210153, on 25/04/2022, before participant enrolment. This study was conducted in accordance with the Declaration of Helsinki and complied with the Clinical Trials Act and its associated regulations.

### Participants

Eligible participants were women aged 20–40 years, classified as American Society of Anaesthesiologists physical status II, with singleton pregnancies at ≥ 37 weeks’ gestation, scheduled for elective CD, able to understand Japanese, and who provided written consent. All parturients received neuraxial anaesthesia using either the spinal or combined spinal-epidural technique. Exclusion criteria included high risk of uterine atony (e.g., uterine fibroids, adenomyosis, endometriotic cyst, macrosomia, polyhydramnios, abnormal placental position, abnormal placenta, uterine malformation, hypertensive disorders of pregnancy), coagulation disorders, spinal conditions that could complicate neuraxial anaesthesia, intrapartum conditions, oxytocin allergy, obesity (body mass index [BMI] ≥ 30 kg/m^2^ at 35–36 weeks’ gestation), previous PPH, in vitro fertilisation pregnancies, diagnosed mental health disorders, or other factors deemed inappropriate by the investigator.

### Randomisation and blinding

Randomisation was performed using a block randomisation method with a block size of 24 to ensure a balance between the groups. Parturients were randomly assigned to one of two groups: the IMY group, which received 5 IU of oxytocin via intramyometrial injection, and the IV group, which received 5 IU of oxytocin in 100 mL of saline via continuous intravenous infusion at 50 mL/h. The allocation sequence was generated by the Clinical Research Centre using Microsoft Excel, with concealment ensured using a scratch-off allocation table. Parturients were enrolled by the attending anaesthesiologist, who was also the principal investigator, and removed the scratch-off layer immediately before assigning the parturients to the interventions. The allocation table was securely stored in a locked cabinet accessible only to the principal investigator, ensuring confidentiality until group assignments were revealed. All medical staff and parturients, except the attending anaesthesiologist, who was also the principal investigator, were blinded to the group assignment. A handheld tissue hardness meter (PEK-MP, Imoto Machinery Co., Ltd, Kyoto, Japan) was used to objectively assess uterine tone^20^, and the evaluator was also blinded to the group assignments. The unblinded principal investigator prepared the blinded study drugs, managed anaesthesia, administered the initial IV bolus of oxytocin and the maintenance infusion of saline (with or without oxytocin), administered phenylephrine, assessed adverse events, and analysed the data. This investigator did not measure blood loss, assess uterine tone (palpation or PEK-MP), determine the need for additional uterotonics or uterine massage, classify refractory uterine atony, perform surgical interventions or blood transfusion, or make decisions regarding hospital discharge. The oxytocin dosage for the IV group was based on international guidelines^9^, and the same dosage was used for the IMY group.

### Anaesthesia, monitoring, and intervention protocols

Upon arrival in the operating theatre, parturients were monitored with electrocardiography, pulse oximetry, and non-invasive blood pressure measurement, as well as a non-invasive continuous haemodynamic monitor (ClearSight system, Edwards Lifesciences Corporation, Irvine, CA, USA). Baseline systolic blood pressure (SBP), mean arterial pressure (MAP), heart rate (HR), cardiac output (CO), and stroke volume (SV) were calculated as the mean of three measurements taken after stabilisation in the supine position. Two peripheral venous routes were secured: one for infusing 500 mL of colloid (Voluven, Otsuka Pharmaceutical Co., Ltd., Tokyo, Japan) completed before surgery, and another for bicarbonate Ringer’s solution (BICANATE, Otsuka Pharmaceutical Co., Ltd., Tokyo, Japan) at 100 mL/h, with the side port reserved exclusively for continuous IV administration of the study drug.

After metoclopramide 10 mg was administered intravenously, neuraxial anaesthesia was induced in the left lateral recumbent position. The puncture was performed at the L3/4 level using a pencil-point needle. Hyperbaric bupivacaine 12 mg, fentanyl 10 µg, and preservative-free morphine 100 µg were administered into the intrathecal space. Following anaesthesia, the parturients were placed in the supine position with left uterine displacement. Continuous IV phenylephrine was administered to maintain SBP between 90 and 120% of baseline, and anaesthesia levels were confirmed to reach Th5 or higher before surgery; cases failing to achieve this were excluded. Continuous IV phenylephrine administration and left uterine displacement were continued until childbirth. After delivery, hypotension, defined as SBP dropping below 80% of the baseline, was treated with 0.1 mg of intravenous phenylephrine.

Immediately after placental delivery, all participants received an initial IV bolus of 1 IU oxytocin over 15 s. This was followed by a group-specific maintenance regimen. The IMY group received 5 IU (1 mL) oxytocin, injected intramyometrically into the uterine fundus, and a continuous IV infusion of 100 mL saline was started at 50 mL/h for 2 h. The IV group received 1 mL saline, injected intramyometrically, and 5 IU oxytocin in 100 mL saline was continuously infused at 50 mL/h for 2 h. IMY injections were administered by obstetricians, and IV infusions were managed by the attending anaesthesiologist. Uterine tone was assessed by obstetricians at 3, 5, 7, 9, 11, 15, and 19 min after administration of the study drugs and categorised as ‘sufficient’ or ‘insufficient’. If the peritoneum was closed and assessment was difficult, that time point was excluded. Uterine fundus hardness was measured by the same assessor using a PEK-MP at 3, 5, and 9 min after the administration of the study drugs as well, and at uterine and peritoneal closure. The obstetrician or midwife measured the distance between the uterine fundus and umbilicus using the width of their fingers immediately after surgery, upon return to the ward, and at 15, 30, 60, and 120 min. The postpartum women were transferred to the ward immediately after leaving the operating theatre.

In cases of inadequate uterine contractions, additional uterotonics were administered according to a specific regimen. If the uterine tone was deemed inadequate, a 1 IU bolus of oxytocin was administered at the obstetrician’s discretion. If a total of ≥ 5 IU of additional oxytocin was required, a different uterotonic (e.g., methylergometrine or dinoprost) could be administered^5^. Surgical interventions, such as compression sutures, balloon tamponade, gauze tamponade, vascular ligation, hysterectomy, or interventional radiology, were performed as necessary, also at the discretion of the obstetrician.

### Primary and secondary outcomes

The primary outcome was the total blood loss from the start of surgery to two hours after returning to the ward. Blood loss was calculated by adding up the fluid aspirated into the suction bottle to the fluid absorbed by the gauze and other materials. After removing the amniotic fluid through a uterine incision, the aspirated blood volume was measured precisely, and blood absorption in the materials was determined by comparing their wet and dry weights^19^. Operating room nurses measured blood loss.

Secondary outcomes included the incidence of total blood loss ≥ 1000 mL and the degree of uterine contractions, assessed both by palpation and with the PEK-MP device. Additionally, the required additional uterotonics, including oxytocin, frequency of uterine massages, and occurrence of refractory uterine atony, were recorded. Refractory uterine atony was defined as cases in which the obstetrician deviated from the protocol due to the ineffectiveness of the protocol uterotonics or when surgical intervention was required. Surgical interventions, blood transfusions, length of hospital stay, and the total phenylephrine dose administered after delivery were also documented.

### Assessment of oxytocin-related adverse effects

The incidence of clinically relevant oxytocin-related adverse effects, such as bradycardia (HR < 40 beats per minute), tachycardia (HR ≥ 120 beats per minute), hypotension (SBP ≤ 80% of baseline), was evaluated. These adverse effects were monitored by an unblinded investigator from the start of oxytocin administration until the end of surgery. Haemodynamic assessment was conducted by evaluating the relative changes in each parameter, including SBP, MAP, HR, CO, and SV. The relative change in each parameter was defined as the value compared with its respective baseline measurement. The time of the study drug administration was defined as T0, and the relative changes in each parameter were assessed at four time points: 5 min (T5), 10 min (T10), 20 min (T20), and 30 min (T30) after the start of administration.

### Statistical analyses

Primary and secondary outcomes and oxytocin-related adverse effects were compared between the groups using the following statistical methods unless otherwise specified. Continuous data were assessed for normal distribution using quantile–quantile plots. Normally distributed data were compared using two-sided unpaired t-tests, whereas non-normally distributed data were analysed using two-sided Mann–Whitney U tests. Categorical data were compared using Fisher’s exact test. Data are presented as the mean (standard deviation), median (interquartile range), or number (percentage). For continuous variables, the median difference between the groups was estimated using the Hodges–Lehmann estimator with 95% confidence intervals. Binary variables were analysed using both absolute and relative effect measures. The absolute effect size was represented by the risk difference with a 95% confidence interval calculated using Newcombe’s method based on Wilson score intervals. The relative effect sizes were represented by the risk ratio and odds ratio.

The time course of the relative changes in haemodynamic parameters was evaluated using two approaches. First, comparisons within each group (IMY group or IV group) between T0 and each time point (T5, T10, T20, and T30) were performed using two-sided paired t-tests or Wilcoxon signed-rank tests depending on data normality. Second, between-group comparisons (IMY group vs. IV group) were conducted at the same four time points (T5, T10, T20, and T30). For both analyses, the Bonferroni correction was applied to account for multiple comparisons, with a significance level of P < 0.0125 considered statistically significant. Uterine contraction was evaluated using palpation intraoperatively on a 7-point scale (P < 0.0072) and postoperatively on a 6-point scale (P < 0.0014), with both significance levels adjusted using Bonferroni correction. Additionally, assessments using the PEK-MP device, based on a 5-point scale, were considered significant at P < 0.01. All other comparisons were considered significant at P < 0.05.

Sample size calculations based on previous data^19^ indicated that 8 parturients per group were required to detect a 100 mL reduction in blood loss with 80% power and at a 5% significance level, using a pooled standard deviation of approximately 70 mL derived from that study and a two-sample t test. A difference of 100 mL was considered clinically meaningful by the obstetricians and anaesthesiologists involved in the study design. To account for potential attrition, 12 parturients were recruited per group, resulting in 24 parturients. The trial concluded that when more than eight effective cases were observed in each group, the minimum required effective sample size was met. Additionally, the trial was designed to terminate only in the event of unexpected severe adverse events. All statistical analyses were performed using R version 4.4.1 (R Foundation for Statistical Computing, Vienna, Austria)^21^.

The Full Analysis Set (FAS) included all randomised parturients, except those who did not undergo CD, withdrew consent, or had missing primary outcome data. The primary and secondary outcomes were analysed using the FAS. For oxytocin-related adverse event analysis, all randomised parturients were included, except those who did not undergo CD or had no available adverse event data.

## Results

### Study population

**Fig. 1:**
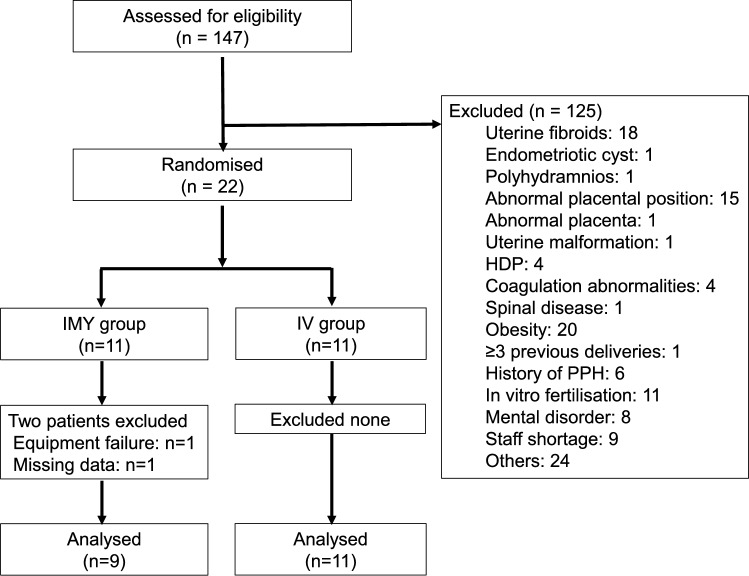
CONSORT diagram. *HDP* hypertensive disorders of pregnancy, *PPH* postpartum haemorrhage, *IMY* intramyometrial, *IV* intravenous.

Between April 2022 and January 2024, a total of 147 women were assessed for eligibility to participate. Of these, 125 were excluded and 22 were randomised into two groups: 11 were assigned to the IMY group and 11 to the IV group. In the IMY group, one parturient was excluded due to a failure of the ClearSight system monitoring before the study commenced, and another was excluded due to missing data on the primary outcome. Consequently, 9 parturients in the IMY group and 11 parturients in the IV group were included in the FAS (n = 20) (Fig. 1). All analyses were conducted according to the originally assigned groups (intention-to-treat analysis). The trial was concluded after achieving the required sample size, as determined by the sample size calculation.

### Efficacy of oxytocin

**Fig. 2:**
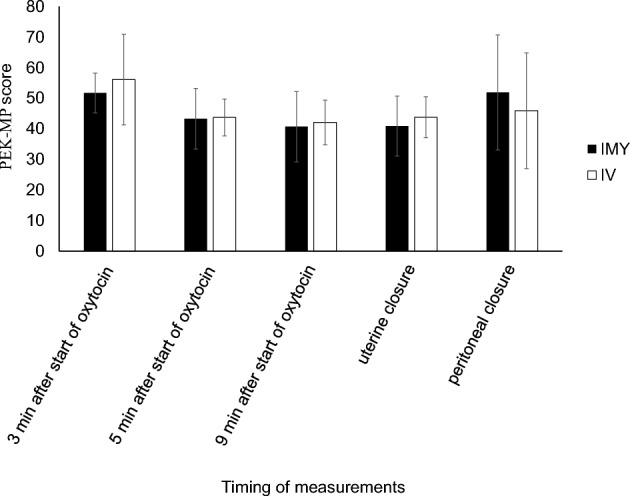
Objective uterine tone measured using the PEK-MP of parturients administered IMY or continuous IV infusion. Values were presented as means (standard deviations). No significant differences were observed between the two groups at any of the measurement points (P < 0.01). *IMY* intramyometrial, *IV* intravenous.

The baseline characteristics of the participants in the FAS were similar between the two groups (Table 1). There were no significant differences in total blood loss between the IMY and IV groups (634 mL [540–718] vs. 642 mL [507.5–706.5], P = 0.66), with a median difference of 1.0 mL (95% CI − 158.0 to 224.0 mL). All secondary outcomes were similar, except for phenylephrine requirements, which were significantly lower in the IMY group (Table 2, Supplementary Table S1). Uterine contractions, as assessed by palpation and the PEK-MP at all time points, also showed no significant differences between the groups (Fig. 2, Tables 3, 4).

**Table 1:** Baseline and obstetric characteristics.

	IMY	IV
	(n = 9)	(n = 11)
Maternal age (y)	31.3 (2.7)	33.6 (5.0)
Height (cm)	157.2 (3.9)	160 (6.1)
Weight (kg)	61.5 (8.5)	60.1 (6.9)
BMI (kg/m^2^)	24.2 (3.3)	23.1 (3.1)
Gestational age (wk)	38.1 (0.5)	37.8 (0.5)
Gravid	2 (2–2)	2 (2–3)
Parity	1 (1–1)	1 (1–1)
Number of prior CDs	1 (1–1)	1 (1–1)
Duration of surgery (min)	66.6 (22.8)	69.7 (12.8)
Duration of anaesthesia (min)	87.0 (25.4)	88.7 (12.7)
Sensory block level		
Left	T4 (T4–T5)	T4 (T3–T5)
Right	T4 (T2–T5)	T4 (T2–T5)
Baseline value of		
SBP (mmHg)	113.4 (14.7)	116.7 (16.4)
MAP (mmHg)	86.0 (9.7)	89.2 (11.2)
HR (beats/min)	77.2 (8.4)	80.2 (14.0)
CO (L/min)	6.5 (1.4)	6.5 (1.0)
SV (mL/min)	84.3 (14.0)	81.6 (11.0)

Continuous data were expressed as means (standard deviation) or medians (interquartile range). *IMY* intramyometrial, *IV* intravenous, *BMI* body mass index, *CD* caesarean delivery, *SBP* systolic blood pressure, *MAP* mean arterial pressure, *HR* heart rate, *CO* cardiac output, *SV* stroke volume.

**Table 2:** Efficacy of oxytocin.

	IMY	IV	P
	(n = 9)	(n = 11)	
Total blood loss (mL)	634 (540–718)	642 (507.5–706.5)	0.66
Intraoperative (mL)	556 (495–619)	551 (430–646.5)	0.62
Postoperative (mL)	40 (30–45)	60 (40–100)	0.25
Total blood loss ≥ 1000 mL	1 (11.1%)	1 (9.1%)	1
Required additional oxytocin	5 (55.5%)	7 (63.6%)	1
Additional oxytocin dose (IU)	1 (0–2)	1 (0–2)	1
Intraoperative (IU)	1 (0–1)	1 (0–1.5)	0.90
Postoperative (IU)	0 (0–0)	0 (0–0.5)	0.84
Additional uterotonic other than oxytocin	1 (11.1%)	0 (0%)	0.45
Required Uterine massage	8 (88.8%)	9 (81.8%)	1
Number of uterine massages	3 (1–4)	2 (1.5–3.5)	0.85
Refractory uterine atony	1 (11.1%)	0 (0%)	0.45
Surgical intervention	0 (0%)	0 (0%)	1
Transfusion	0 (0%)	0 (0%)	1
Length of hospital stay (days)	7 (7–8)	7 (7–8)	0.38
Total phenylephrine dose after delivery (mg)	0.2 (0.1–0.3)	0.6 (0.4–0.6)	0.02

Continuous data were expressed as medians (interquartile range), and dichotomous data were expressed as n (%). *IMY* intramyometrial, *IV* intravenous, *IU* international units, *N/A* Not Applicable.

**Table 3:** Percentage of adequate uterine contractions.

	IMY	IV	P
	(n = 9)	(n = 11)	
Time after start of oxytocin			
3 min	8/9 (88.8%)	11/11 (100%)	0.45
5 min	8/9 (88.8%)	10/11 (90.9%)	1
7 min	7/9 (77.7%)	11/11 (100%)	0.19
9 min	7/9 (77.7%)	8/11 (72.7%)	1
11 min	7/9 (77.7%)	10/11 (90.9%)	0.57
15 min	8/9 (88.8%)	10/11 (90.9)	1
19 min	8/8 (100%)	9/11 (81.8%)	0.49

Values were expressed as n (%). *IMY* intramyometrial, *IV* intravenous.

**Table 4:** Finger width from below the umbilicus to the fundus of the uterus after surgery.

	IMY	IV	P
	(n = 9)	(n = 11)	
At the end of surgery	2 (1–2)	2 (2–3)	0.36
Immediately on return to the ward	1 (1–2)	2 (1–2)	0.66
15 min after return to the ward	2 (1–2)	1 (1–2.5)	0.91
30 min after return to the ward	2 (1–2)	1 (1–2)	0.94
60 min after return to the ward	1 (1–2)	2 (0.5–2.5)	0.53
120 min after return to the ward	1 (1–2)	2 (1–3)	0.21

Values were presented as medians (interquartile range). *IMY* intramyometrial, *IV* intravenous.

### Assessment of oxytocin-related adverse effects

Adverse effects were analysed in 21 of the 22 randomised parturients: 10 in the IMY group and 11 in the IV group. Hypotension was observed in 8 of 10 parturients in the IMY group and in all parturients in the IV group, with no significant difference in the frequency of hypotension between the groups. Similarly, no significant differences were observed in the incidence of other adverse events (Table 5). No serious adverse events were reported in either group and all adverse events resolved within the same day.

**Table 5:** Oxytocin-induced adverse effects.

	IMY	IV	P
	(n = 10)	(n = 11)	
Hypotension	8 (80%)	11 (100%)	0.21
Hypertension	0 (0%)	1 (9.1%)	1
Bradycardia	0 (0%)	0 (0%)	1
Tachycardia	0 (0%)	0 (0%)	1
Flushing	0 (0%)	3 (27.3%)	0.21
Chest pain	0 (0%)	0 (0%)	1
Dyspnoea	5 (50%)	2 (18.2%)	0.18
Headache	2 (20%)	0 (0%)	0.21
ST change	1 (10%)	0 (0%)	0.48
Nausea and vomiting	3 (30%)	1 (9.1%)	0.31

Values were presented as n (%). *IMY* intramyometrial, *IV* intravenous.

**Fig. 3:**
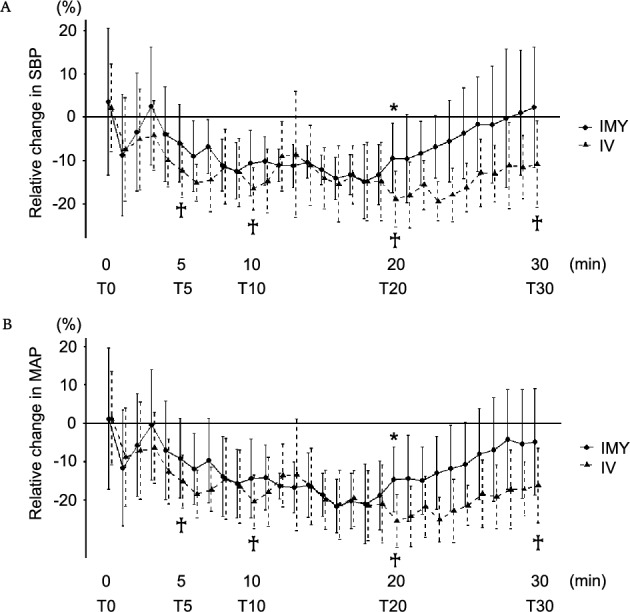
Time course of relative haemodynamic changes in parturients administered IMY or IV oxytocin. Relative changes in systolic blood pressure (SBP) (**A**) and mean arterial pressure (MAP) (**B**) were defined as the percentage change relative to baseline upon entry into the operating room. T0 indicates the oxytocin injection time. Values were presented as the means (standard deviation). Relative changes were evaluated at T5, T10, T20, and T30. *P < 0.0125, IMY group vs. IV group; ✟P < 0.0125, compared with T0 within group. *IMY* intramyometrial, *IV* intravenous.

Time-course analysis revealed that the relative changes in SBP and MAP at T20 were significantly greater in the IMY group than those in the IV group. Within the IV group, the relative changes in SBP and MAP showed significantly larger negative deviations at T5, T10, T20, and T30 than at T0. In contrast, within the IMY group, the relative changes in SBP and MAP showed no significant deviations from T0 across all assessed time points (Fig. 3). No significant differences were observed in the relative changes in HR, CO, or SV between the groups, or relative to the values at T0 (Supplementary Fig. S1).

## Discussion

This study suggested that after all participants received an initial IV bolus of 1 IU oxytocin following placental delivery, total blood loss did not differ significantly between women receiving a 5 IU IMY oxytocin injection and those receiving a continuous IV infusion of 5 IU oxytocin as a maintenance regimen to sustain uterine contractility during elective CD in this small, low-risk elective cohort. Measures of uterine tone and the need for additional interventions were also similar between groups. However, IMY administration was associated with fewer haemodynamic fluctuations and reduced phenylephrine requirements, consistent with improved haemodynamic stability.

Prevention of PPH during CD involves two phases of oxytocin administration: an initial dose to induce uterine contractions and a maintenance dose to sustain them. Previous studies have primarily focused on the initial dose and its effectiveness in inducing adequate uterine contractions^10,11,19,22–24^. However, the importance of maintenance dose has also been reported. Specifically, studies of continuous IV infusion have shown that a maintenance dose reduces blood loss^14,15^, the need for additional oxytocin^14,15^, and the frequency of blood transfusions^15^.

Although IMY oxytocin has been less commonly studied, several studies have focused on its initial dose. Compared to IV administration, IMY oxytocin has a slower onset^19^, and uterine contractility has been shown to be comparable when IMY is administered at four times the intravenous dose^22^. However, its role as a maintenance dose has not been fully explored. In the present study, no significant difference in total blood loss was observed between IMY and IV infusions. This can be attributed to several factors. Although no studies have directly measured oxytocin concentrations after IMY administration, previous research on intramuscular oxytocin has shown that blood concentrations peak at 10 min and decline within approximately 60 minutes^25^. In contrast, the continuous IV infusion maintained a steady blood concentration of oxytocin^26^. It is possible that the IMY injection temporarily increases oxytocin concentrations locally within the uterine muscle, thereby enhancing uterine contractility. However, this effect may weaken after approximately 60 min, resulting in blood loss comparable to that observed with intravenous infusion.

In the present study, IMY oxytocin administration resulted in reduced blood pressure variability and lower phenylephrine requirements than IV infusion. Our previous study similarly demonstrated that IMY oxytocin maintained a higher blood pressure three minutes after administration than after IV injection^19^. Although the pharmacokinetics of IMY oxytocin have not been directly studied, the observed haemodynamic differences may be explained by differences in the increase in oxytocin blood concentrations. IMY administration, which involves direct injection into the uterine muscle, may lead to a slower and more gradual increase in systemic oxytocin concentrations than the steady-state concentrations achieved through continuous IV infusion^25,26^. This gradual increase in oxytocin levels could reduce the likelihood of acute haemodynamic changes, thereby contributing to the haemodynamic stability observed in the IMY group. Such haemodynamic stability may be advantageous for parturients with cardiovascular comorbidities. However, this study was not designed or powered to evaluate management of major haemorrhage; in such situations, escalation according to institutional protocols—including additional IV oxytocin boluses and/or second-line uterotonics and surgical measures—remains essential^9^.

This study had two strengths. First, uterine tone was objectively measured using a hardness meter, providing a more reliable and consistent assessment than traditional subjective evaluations by obstetricians. Recent research demonstrated that objective measurements using a hardness meter correlate well with subjective assessments and intraoperative blood loss, thereby enhancing the accuracy of uterine tone evaluation in clinical practice^20^. Second, the study incorporated detailed haemodynamic monitoring using ClearSight system technology, allowing for a comprehensive analysis of cardiovascular stability through continuous measurement of SBP, MAP, HR, CO, and SV following oxytocin administration. To the best of our knowledge, this is the first study to precisely evaluate haemodynamic parameters during IMY injection of oxytocin or continuous intravenous oxytocin infusion, as recommended by the International Consensus Statement^9^.

However, this study had several limitations. First, it focused on low-risk women undergoing elective CD at a single tertiary care centre. Therefore, these findings may not be generalisable to higher-risk populations, such as those undergoing intrapartum CD, who may require higher oxytocin doses for adequate uterine tone^10,27,28^. Further research in diverse settings with a broader parturient population is required to confirm these results. Second, the potential impact of vasopressors and uterotonics on ClearSight system measurements was not explicitly evaluated. Phenylephrine increases systemic vascular resistance (SVR), which potentially affects CO accuracy^29^. Previous studies have shown that the ClearSight system provides acceptable accuracy in measuring blood pressure but has limited reliability for CO measurements, particularly in patients with altered SVR^29^. Future studies should adjust for these effects to improve non-invasive haemodynamic assessment accuracy. Third, total blood loss was measured only up to 2 h postoperatively, reflecting the expected duration of the effects of oxytocin after IMY injections ^25^. Future studies should extend this monitoring period to evaluate long-term outcomes and capture later bleeding events, including delayed postpartum haemorrhage. Fourth, data analysis was performed by an unblinded principal investigator, which represents a potential source of bias. To minimise this risk, exclusions were made strictly according to a predefined protocol, and statistical analyses were conducted according to pre-specified methods. Finally, the sample size calculation was based on a previous study that used IMY injection for the initial oxytocin administration^19^, which differs from the protocol used in this study. Future research should calculate the sample size based on the current protocol to improve the accuracy. In addition, the sample size was small and the trial was not powered to detect differences in rare but clinically important outcomes, such as major postpartum haemorrhage, transfusion, or surgical haemostasis. Therefore, the findings should be interpreted primarily in terms of short-term blood loss and haemodynamic profiles in a low-risk elective population.

In this small, low-risk elective cohort, total blood loss did not differ significantly between the IMY and IV groups. However, IMY administration was associated with better haemodynamic stability**.** In cases of major haemorrhage, escalation according to standard IV protocols and additional uterotonics remains essential. Further studies involving various parturient populations are required to validate and expand upon these findings.

In conclusion, in the prevention of PPH during elective CD, both groups received an initial IV bolus of 1 IU oxytocin. A maintenance dose of 5 IU IMY oxytocin did not result in a significant difference in total blood loss from the intraoperative period to two hours postoperatively compared with continuous IV infusion of 5 IU. However, IMY injection was associated with better haemodynamic stability in this small, low-risk elective cohort. Future studies should focus on high-risk populations, such as parturients with cardiovascular comorbidities or significant haemorrhage risk, and investigate the pharmacokinetics of IMY oxytocin, including its blood concentration profile, following administration.

## Supplementary Information

Supplementary Information.

## Data Availability

The datasets used and/or analysed in this study are available from the corresponding author upon reasonable request. The details of the trial protocol can be found in the jRCT registration record (jRCTs041210153) at the following URL: https://jrct.mhlw.go.jp/latest-detail/jRCTs041210153.
