# Supplementary material for: Intramyometrial injection versus intravenous infusion of oxytocin for maintaining uterine contractility during elective caesarean delivery in a randomised controlled trial
Source: Sci Rep. 2026 Mar 31;16:15571. doi: 10.1038/s41598-026-46727-z (PMC13186971; doi:10.1038/s41598-026-46727-z)
Supplement: Supplementary file 1 — Supplementary Information. [file 41598_2026_46727_MOESM1_ESM.pdf]

Title:

Intramyometrial injection versus intravenous infusion of oxytocin for maintaining uterine contractility during elective caesarean delivery in a randomised controlled trial

Authors:

Satoshi Naruse, Chieko Akinaga, Yusuke Mazda, Yoshiki Nakajima

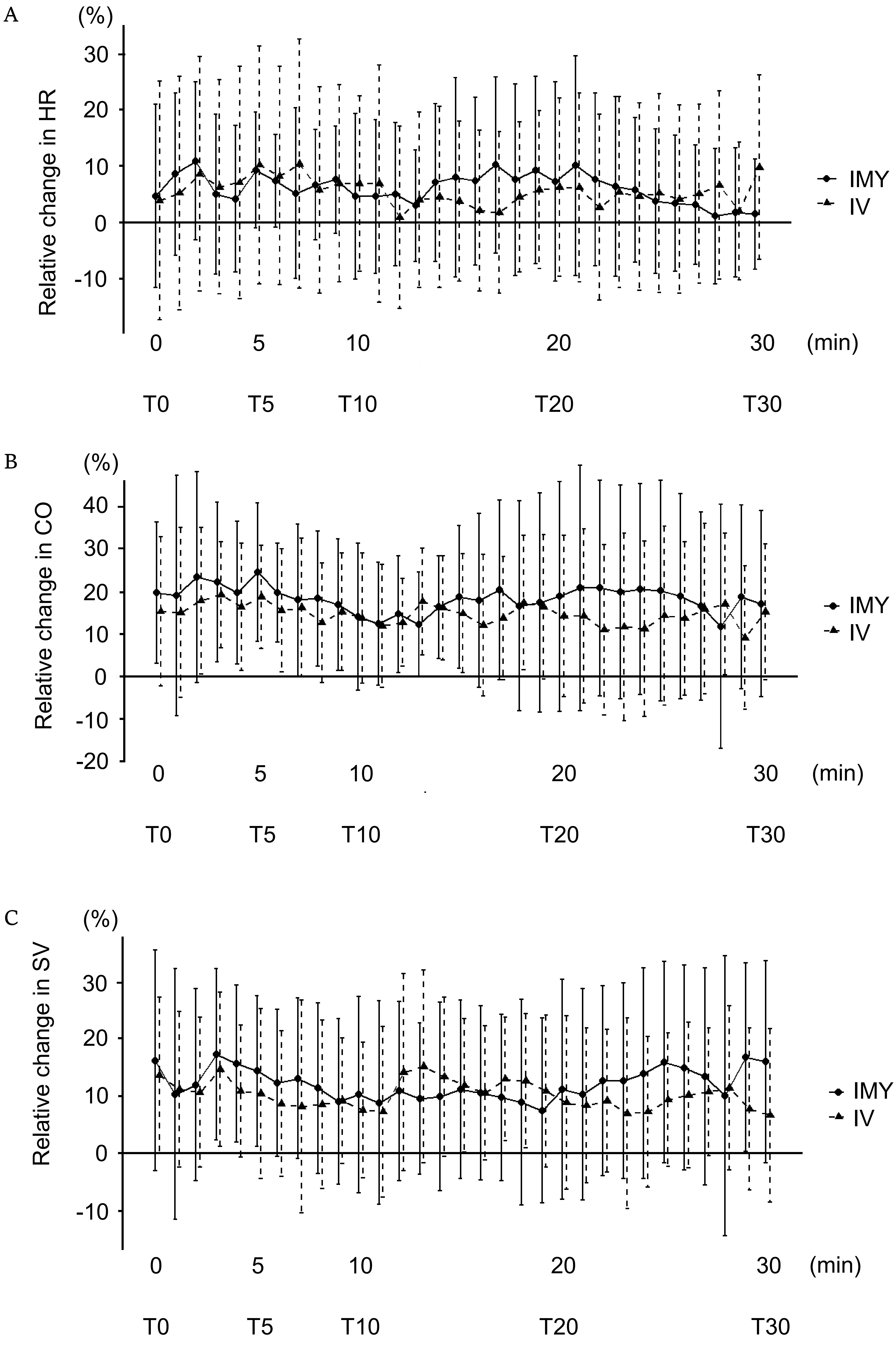

Supplementary Fig. S1. Time course of relative haemodynamic changes in parturients given IMY or IV oxytocin

Relative changes in heart rate (HR) (A), cardiac output (CO) (B), and stroke volume (SV) (C) were defined as the percentage change relative to baseline upon entry to the operating room. T0 indicates the moment of oxytocin injection. Values were presented as means (standard deviation). No significant differences were observed either within each group (IMY group or IV group) compared to T0, or between the IMY and IV groups at any time point (T5, T10, T20, T30). IMY, intramyometrial; IV, intravenous.

Supplementary Table S1. Additional Outcomes of Oxytocin Efficacy with Median, Risk, and Odds Ratios

	IMY (n=9)	IV (n=11)	Median Difference (95% CI)	Risk Difference (95% CI)	Odds Ratio (95% CI)	P
Total blood loss (mL)	634 (540–718)	642 (507.5–706.5)	1.0 (-158.0–224.0)			0.66
Intraoperative (mL)	556 (495–619)	551 (430–646.5)	47.0 (-94.0–244.0)			0.62
Postoperative (mL)	40 (30–45)	60 (40–100)	-15.0 (-60.0–25.0)			0.25
Total blood loss ≥1,000 mL	1 (11.1%)	1 (9.1%)		2.0% (-41.2%–45.3%)	1.2 (0.0–107.7)	1
Required additional oxytocin	5 (55.5%)	7 (63.6%)		-8.3% (-54.3%–37.7%)	0.7 (0.2–11.9)	1
Additional oxytocin dose (IU)	1 (0–2)	1 (0–2)	0.0 (-1.0–1.0)			1
Intraoperative (IU)	1 (0–1)	1 (0–1.5)	0.0 (-1.0–1.0)			0.90
Postoperative (IU)	0 (0–0)	0 (0–0.5)	0.0 (-0.0–0.0)			0.84
Additional uterotonic other than oxytocin	1 (11.1%)	0 (0%)		8.3% (-15.0%–31.7%)	N/A	0.45
Required Uterine massage	8 (88.8%)	9 (81.8%)		2.4% (-36.6%–41.4%)	0.6 (0.0–13.2)	1
Number of uterine massages	3 (1–4)	2 (1.5–3.5)	0.0 (-2.0–2.0)			0.85
Refractory uterine atony	1 (11.1%)	0 (0%)		8.3% (-15.0%–31.7%)	N/A	0.45
Surgical intervention	0 (0%)	0 (0%)		N/A	N/A	1
Transfusion	0 (0%)	0 (0%)		N/A	N/A	1
Length of hospital stay (day)	7 (7–8)	7 (7–8)	0.0 (-0.0–1.0)			0.38
Total phenylephrine dose after delivery (mg)	0.2 (0.1–0.3)	0.6 (0.4–0.6)	-0.30 (-0.50–-0.10)			0.02

Continuous data were expressed as medians (interquartile range), and dichotomous data were expressed as n (%). Median Difference and Risk Difference were presented with 95% confidence intervals (CI). IMY, intramyometrial; IV, intravenous; IU, international units; N/A, Not Applicable.
